# Supplementary material for: Persistence and In Vivo Evolution of Vaginal Bacterial Strains over a Multiyear Time Period
Source: mSystems. 2022 Nov 22;7(6):e00893-22. doi: 10.1128/msystems.00893-22 (PMC9764964; doi:10.1128/msystems.00893-22)
Supplement: TEXT S1 [file msystems.00893-22-s0001.docx]

## Supplemental Text 1

DNA extraction & Library prep

Whole genomic DNA was extracted from 500µl aliquot of re-suspended Amies Eswab solution as described before (1, 2). The vaginal swab suspensions were lysed using a mixture which contained three enzymes: 50 μl of lyzosyme (10 mg/ml), 6 μl of mutanolysin (25,000 U/ml Sigma- Aldrich), 3 μl of lysostaphin (4000 U/ml in sodium acetate; Sigma- Aldrich), and 41 μl of TE50 buffer (10 mM Tris·HCL and 50 mM EDTA, pH 8.0). The mixture was incubated at 37 °C for 1 hour and then bead beating (0.1-mm-diameter zirconia/silica beads, BioSpec Products) was conducted for 1 min using a Mini-Beadbeater-96 operating at 2100rpm (BioSpec Products). DNA was purified from this lysate using the QIAamp DNA Mini Kit (Qiagen) and then eluted using 200 μl of TE buffer (10 mM Tris-Cl, 0.5 mM EDTA; pH 9.0). Sequence libraries were then constructed using Illumina Nextera XT kits and sequenced using an Illumina HiSeq 2500 (100 bp paired end mode) at the Institute for Genome Sciences in the University of Maryland School of Medicine.

Bioinformatics processing

Sequencing generated an average of X read pairs per metagenome (range: ). Host reads were removed using BMTagger (v3.101) in combination with the human genome reference (GRCh37.p5). Ribosomal RNA sequence reads were removed based on the Bowtie (3) mapping results to the SILVA reference database (4). Both read pairs were filtered out if at least one read mapped to either the human genome or the rRNA database. Raw reads with low-quality bases as determined by base calling (phred quality score of 20) were trimmed from the end of the sequence, and only sequence longer than 75bp were retained. Reads were then mapped to the VIRGO non-redundant gene catalog (5) to establish taxonomic composition using Bowtie. The relative abundances of each bacterium was calculated after correcting for gene length, as described previously (6). De novo assembly was then performed using metaspades with default settings (7, 8). The resulting contigs were then binned based on reads mapping results to the VIRGO non-redundant gene catalog, as described previously (9). Quality of the metagenome assembled genomes (MAGs) was assessed using checkM (10) and only MAGs demonstrating a contamination level less than 5% and a completeness greater than 95% were included in the analysis.

## Construction of the phylogenetic tree

The genes encoded in each MAG were identified using PROKKA (11) and orthologous genes were identified using orthoMCL as described previously (12). Orthologous genes present in at least 98% of the MAGs were used to establish the phylogenetic tree (n=100 genes). Each orthologous gene set was first aligned using Muscle (13) and then the alignments were concatenated using phyutility (14). An appropriate partitioning scheme and model of molecular evolution was identified using PartitionFinder (15). The phylogeny was then established using RaxML-ng (16) with 20 starting trees (10 based on parsimony and 10 randomly generated). Bootstraps converged after 250 replicates as detected using the autoMRE setting.

Identification of persistent strains and mutation characterization

Persistent strains were identified using the inStrain tool (17). Each metagenome’s reads were mapped to a database comprised of the 53 MAGs to generate inStrain profiles using Bowtie2 (18). The inStrain profiles were then summarized using the inStrain compare function which provided overlap and sequence similarity between 53 MAGs and each metagenome’s sequence reads. Persistent strains were then identified using a stringent sequence similarity threshold (50% coverage and at least 99.999% population ANI). For each persistent strain demonstrating at least 15X average coverage, mutations were identified with BreSeq (19) based on the mapping of the sequence reads from the second timepoint (T_2_) to the MAG generated using the initial timepoint’s metagenome (T_1_). To minimize the effect of mis-mapped reads originating from related strains in the same metagenome, competitive mapping was first used to subset out only the focal strain’s sequence reads from entire the metagenome dataset (Bowtie (20), settings: -l 25 --fullref --best --strata -m 20). These subset reads were then mapped to the T_0_ MAG using Breseq (v.332, default settings). The median coverage of identified variants was 42x (range: 4x-544x) and their median frequency in the population of 94% (range 80%-100%).

STORMS checklist available at: https://github.com/ravel-lab/Two_year.

## References

1. Yuan S, Cohen DB, Ravel J, Abdo Z, Forney LJ. 2012. Evaluation of methods for the extraction and purification of DNA from the human microbiome. PLoS One 7:e33865.

2. Ravel J, Gajer P, Abdo Z, Schneider GM, Koenig SS, McCulle SL, Karlebach S, Gorle R, Russell J, Tacket CO, Brotman RM, Davis CC, Ault K, Peralta L, Forney LJ. 2011. Vaginal microbiome of reproductive-age women. Proc Natl Acad Sci USA 108 Suppl 1:4680-7.

3. Langmead B, Trapnell C, Pop M, Salzberg SL. 2009. Ultrafast and memory-efficient alignment of short DNA sequences to the human genome. Genome biology 10:R25.

4. Pruesse E, Quast C, Knittel K, Fuchs BM, Ludwig W, Peplies J, Glockner FO. 2007. SILVA: a comprehensive online resource for quality checked and aligned ribosomal RNA sequence data compatible with ARB. Nucleic acids research 35:7188-96.

5. Ma B, France MT, Crabtree J, Holm JB, Humphrys MS, Brotman RM, Ravel J. 2020. A comprehensive non-redundant gene catalog reveals extensive within-community intraspecies diversity in the human vagina. Nat Commun 11:940.

6. France MT, Fu L, Rutt L, Yang H, Humphrys MS, Narina S, Gajer PM, Ma B, Forney LJ, Ravel J. 2022. Insight into the ecology of vaginal bacteria through integrative analyses of metagenomic and metatranscriptomic data. Genome Biol 23:66.

7. Bankevich A, Nurk S, Antipov D, Gurevich AA, Dvorkin M, Kulikov AS, Lesin VM, Nikolenko SI, Pham S, Prjibelski AD, Pyshkin AV, Sirotkin AV, Vyahhi N, Tesler G, Alekseyev MA, Pevzner PA. 2012. SPAdes: a new genome assembly algorithm and its applications to single-cell sequencing. J Comput Biol 19:455-77.

8. Nurk S, Meleshko D, Korobeynikov A, Pevzner PA. 2017. metaSPAdes: a new versatile metagenomic assembler. Genome Res 27:824-834.

9. France M, Brown S, Rompalo A, Brotman RM, Ravel J. 2021. Identification of shared bacterial strains in the vaginal microbiota of reproductive-age mothers and daughters using genome-resolved metagenomics. bioRvix doi:10.1101/2021.11.16.468914.

10. Parks DH, Imelfort M, Skennerton CT, Hugenholtz P, Tyson GW. 2015. CheckM: assessing the quality of microbial genomes recovered from isolates, single cells, and metagenomes. Genome Res 25:1043-55.

11. Seemann T. 2014. Prokka: Rapid prokaryotic genome annotation, vol 30, p 2068-2069.

12. Li L. 2003. OrthoMCL: Identification of Ortholog Groups for Eukaryotic Genomes. Genome Research 13:2178-2189.

13. Edgar RC. 2004. MUSCLE: a multiple sequence alignment method with reduced time and space complexity. BMC Bioinformatics 5:113.

14. Smith SA, Dunn CW. 2008. Phyutility: A phyloinformatics tool for trees, alignments and molecular data, vol 24, p 715-716.

15. Lanfear R, Calcott B, Ho SY, Guindon S. 2012. Partitionfinder: combined selection of partitioning schemes and substitution models for phylogenetic analyses. Mol Biol Evol 29:1695-701.

16. Kozlov AM, Darriba D, Flouri T, Morel B, Stamatakis A. 2019. RAxML-NG: a fast, scalable and user-friendly tool for maximum likelihood phylogenetic inference. Bioinformatics 35:4453-4455.

17. Olm MR, Crits-Christoph A, Bouma-Gregson K, Firek BA, Morowitz MJ, Banfield JF. 2021. inStrain profiles population microdiversity from metagenomic data and sensitively detects shared microbial strains. Nat Biotechnol 39:727-736.

18. Langmead B, Salzberg SL. 2012. Fast gapped-read alignment with Bowtie 2, vol 9, p 357-359. Nature Publishing Group.

19. Deatherage D, Barrick J. 2014. Identification of mutations in laboratory-evolved microbes from next-generation sequencing data using breseq, vol 1151, p 165-188.

20. Langmead B, Trapnell C, Pop M, Salzberg SL. 2009. Ultrafast and memory-efficient alignment of short DNA sequences to the human genome. Genome Biol 10:R25.
